# Explainable Machine Learning Model for Predicting First-Time Acute Exacerbation in Patients with Chronic Obstructive Pulmonary Disease

**DOI:** 10.3390/jpm12020228

**Published:** 2022-02-07

**Authors:** Chew-Teng Kor, Yi-Rong Li, Pei-Ru Lin, Sheng-Hao Lin, Bing-Yen Wang, Ching-Hsiung Lin

**Affiliations:** 1 Big Data Center, Changhua Christian Hospital, Changhua 500, Taiwan; 179297@cch.org.tw (C.-T.K.); 183778@cch.org.tw (P.-R.L.); 2 Graduate Institute of Statistics and Information Science, National Changhua University of Education, Changhua 500, Taiwan; 3 Thoracic Medicine Research Center, Changhua Christian Hospital, Changhua 500, Taiwan; 181065@cch.org.tw (Y.-R.L.); 112364@cch.org.tw (S.-H.L.); 4 Division of Chest Medicine, Department of Internal Medicine, Changhua Christian Hospital, Changhua 500, Taiwan; 5 Division of Thoracic Surgery, Department of Surgery, Changhua Christian Hospital, Changhua 500, Taiwan; 156283@cch.org.tw; 6 Institute of Genomics and Bioinformatics, National Chung Hsing University, Taichung 402, Taiwan; 7 Department of Recreation and Holistic Wellness, MingDao University, Changhua 523, Taiwan; 8 Artificial Intelligence Development Center, Changhua Christian Hospital, Changhua 500, Taiwan

**Keywords:** COPD, acute exacerbation, explainable machine learning, SHapley Additive exPlanations (SHAP), local explanation

## Abstract

Background: The study developed accurate explainable machine learning (ML) models for predicting first-time acute exacerbation of chronic obstructive pulmonary disease (COPD, AECOPD) at an individual level. Methods: We conducted a retrospective case–control study. A total of 606 patients with COPD were screened for eligibility using registry data from the COPD Pay-for-Performance Program (COPD P4P program) database at Changhua Christian Hospital between January 2017 and December 2019. Recursive feature elimination technology was used to select the optimal subset of features for predicting the occurrence of AECOPD. We developed four ML models to predict first-time AECOPD, and the highest-performing model was applied. Finally, an explainable approach based on ML and the SHapley Additive exPlanations (SHAP) and a local explanation method were used to evaluate the risk of AECOPD and to generate individual explanations of the model’s decisions. Results: The gradient boosting machine (GBM) and support vector machine (SVM) models exhibited superior discrimination ability (area under curve [AUC] = 0.833 [95% confidence interval (CI) 0.745–0.921] and AUC = 0.836 [95% CI 0.757–0.915], respectively). The decision curve analysis indicated that the GBM model exhibited a higher net benefit in distinguishing patients at high risk for AECOPD when the threshold probability was <0.55. The COPD Assessment Test (CAT) and the symptom of wheezing were the two most important features and exhibited the highest SHAP values, followed by monocyte count and white blood cell (WBC) count, coughing, red blood cell (RBC) count, breathing rate, oral long-acting bronchodilator use, chronic pulmonary disease (CPD), systolic blood pressure (SBP), and others. Higher CAT score; monocyte, WBC, and RBC counts; BMI; diastolic blood pressure (DBP); neutrophil-to-lymphocyte ratio; and eosinophil and lymphocyte counts were associated with AECOPD. The presence of symptoms (wheezing, dyspnea, coughing), chronic disease (CPD, congestive heart failure [CHF], sleep disorders, and pneumonia), and use of COPD medications (triple-therapy long-acting bronchodilators, short-acting bronchodilators, oral long-acting bronchodilators, and antibiotics) were also positively associated with AECOPD. A high breathing rate, heart rate, or systolic blood pressure and methylxanthine use were negatively correlated with AECOPD. Conclusions: The ML model was able to accurately assess the risk of AECOPD. The ML model combined with SHAP and the local explanation method were able to provide interpretable and visual explanations of individualized risk predictions, which may assist clinical physicians in understanding the effects of key features in the model and the model’s decision-making process.

## 1. Introduction

Chronic obstructive pulmonary disease (COPD) is the third leading cause of mortality worldwide and imposes a substantial burden on health care systems, primarily because of the occurrence of acute exacerbation [1,2]. Acute exacerbation of COPD (AECOPD) is defined as the substantial worsening of respiratory symptoms in excess of normal day-to-day variations, thus requiring additional therapy [3]. AECOPD increases a patient’s risk of mortality, accelerates the decline of pulmonary functions, harms a patient’s general health, and impairs activities of daily living; it is also a main driver of health care use, such as emergency department visits and hospitalizations [4,5]. In addition, studies have reported that a patient’s prognosis is poor after their first hospital admission for AECOPD [6,7]. Moreover, nonlower respiratory serious adverse events are more frequent after a patient’s first exacerbation [8]. Therefore, establishing accurate methods for predicting exacerbation risk to identify patients at high risk for AECOPD is imperative because such predictions can be valuable in the timely initiation of preventive measures and in facilitating early treatment in clinical settings.

In clinical practice, the strongest identified risk factor for future AECOPD is a history of prior exacerbations; however, the use of this factor as an indicator is clinically restricted due to heterogeneity in patients’ risk of AECOPD [3]. Moreover, this risk factor cannot be used to predict a patient’s first exacerbation [9]. Machine learning (ML) algorithms have recently been used to predict AECOPD by using the real-world data available in electronic medical records (EMRs) or health care administrative data [5,10]. These ML models have exhibited predictive abilities superior to those of a reference model that considered only patient history of AECOPD as a risk factor, which affected the model’s discrimination and calibration [11]. Despite the promising performance of ML in previous studies, evidence of the ability of explainable risk prediction models to assist in disease prognosis is limited [12]. Due to the black-box nature of ML algorithms, explaining the logic behind predictions is often difficult [13]. The lack of interpretability of ML models is a major obstacle to implementation of ML in the medical field [14]. Although several ML models for predicting the onset of AECOPD events have been developed, research on interpretable and personalized models for predicting the risk of first-time AECOPD is still scarce.

In the present study, we established an accurate explainable prediction model to predict first-time AECOPD at an individual level. We combined the ML algorithm with a framework based on SHapley Additive exPlanations (SHAP) and local explanation to provide both algorithmic and biological explainability; our approach can yield a personalized risk profile for each patient to help physicians to optimize their decision-making with respect to COPD exacerbation prevention and to tailor treatment regimens to the individual needs of patients.

## 2. Materials and Methods

### 2.1. Study Participants

This retrospective case–control study was conducted at Changhua Christian Hospital (CCH) in Changhua, Taiwan. A total of 606 patients with COPD were screened for eligibility using registry data from the COPD Pay-for-Performance Program (COPD P4P program) database at CCH between January 2017 and December 2019. The COPD P4P program provides standardized comprehensive COPD care including COPD assessments, modified Medical Research Council (mMRC) dyspnea scores, clinical symptom evaluations, physical examinations, laboratory evaluations, and COPD management education (including that related to smoking cessation, pulmonary rehabilitation, and medication). Comprehensive COPD care is delivered by a coordinated multidisciplinary team comprising physicians, respiratory therapists, and registered nurses specialized in COPD care. A detailed description of the program has been reported in a previous paper [15]. COPD diagnosis was based on the criteria established by the Taiwan Society of Pulmonary and Critical Care Medicine; whether patients met the criteria was determined on the basis of International Classification of Diseases, Tenth Revision (ICD-10) diagnostic codes J41–44, with confirmation made through spirometry (postbronchodilator forced expiratory volume at 1 s [FEV1]/forced vital capacity [FVC] < 70%) during the 90-day period when patients received outpatient care from CCH.

**Figure 1:**
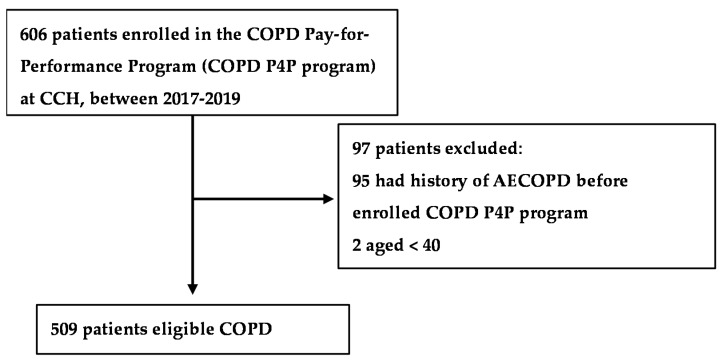
Flowchart of the study population.

Patients with a history of AECOPD before enrollment in the COPD P4P program (*n* = 95) and those younger than 40 years (*n* = 2) were excluded. Ultimately, 509 eligible patients were included in our analysis (Figure 1). We first provided a seed number (1234 was used in this study) for the random number generator in the software R programming, and then performed the “sample” function in R to split the data frame into training and test data. We split the data into 80% training dataset and 20% testing dataset. Finally, patients were randomly divided into a training set (80%, *n* = 407) to develop ML models and a test set (20%, *n* = 102) to evaluate the performance of each model. The Institutional Review Board of CCH waived the requirement for informed consent and approved the study (IRB No: 191246). Data in the accessed database were deidentified. The researchers conducted the study in accordance with the Computer-Processed Personal Data Protection Law and privacy regulations of Taiwan.

### 2.2. Outcome

The study outcome was to early predict the first-time AECOPD after enrollment in a P4P program, which is defined as outpatient visits, emergency room visit, or admission with an ICD-10 code of COPD (J43.x–44.x, except J430, within the fifth secondary diagnosis in outpatient or emergency room visits or as the primary diagnosis upon hospital admission) at which time systemic steroid medication (ATC code: H02) with or without antibiotics (ATC code: J01) was prescribed [10,16].

### 2.3. Feature Engineering

The features were obtained from the CCH clinical research database, which is a collection of data and databases from all the CCH EMR systems, including the COPD P4P database, prescription data, laboratory data, and clinical visit records. We collected 90 features from the outpatient clinical records dated within six months before a patient’s last visit prior to their first AECOPD; these features included demographics (age, gender, BMI), clinical characteristics (e.g., postbronchodilator test results, CAT scores, mMRC dyspnea scores, COPD Global Initiative for Chronic Obstructive Lung Disease [GOLD] scores, respiratory symptoms), vital signs, laboratory test measurements, medication use, and comorbidities. To ensure the variability of features and improve the predictive accuracy of the models [17], 32 features with a prevalence <5% were excluded from the model. In addition, we used recursive feature elimination (RFE) technology to select the optimal subset of features for predicting the occurrence of AECOPD. In total, 38 features were selected using RFE with ten-fold cross-validation repeated five times (Figure S1). Of the 38 features, the postbronchodilator FEV1/FVC ratio, eosinophil-to-lymphocyte ratio, hemoglobin, and COPD GOLD score were excluded for collinearity (variance inflation factor > 2); the number of included features was thereby reduced to 34 after consulting with expert COPD physicians. The descriptive statistics of the selected features for the training set and test set are listed in Table 1.

### 2.4. Statistical Analysis and ML Algorithms

The categorical and continuous variables are expressed herein as proportions and means ± standard deviations, respectively. A chi-square test was used to compare the categorical variables, and Student’s *t*-test was used to compare the continuous variables.

**Figure 2:**
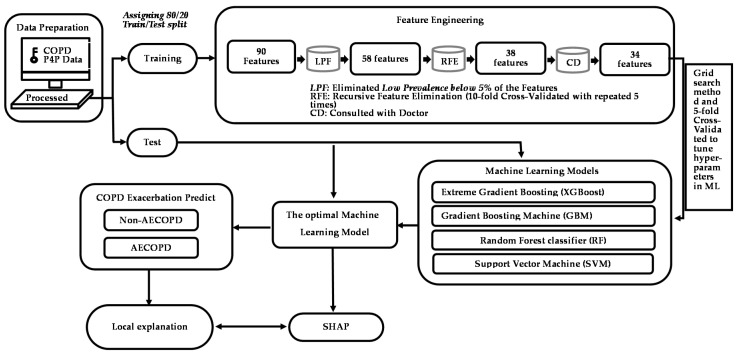
Study framework for AECOPD risk assessment model and feature engineering.

Figure 2 presents the framework for establishing the predictive models for first-time AECOPD, including data preprocessing, feature engineering, ML model construction, and model training. Four ML algorithms—support vector machine (SVM), random forest (RF), gradient boosting machine (GBM), and extreme gradient boosting (XGB)—were used to develop the models. The exhaustive grid search algorithm was implemented as a hyperparameter tuning tool (Table S1), and five-fold cross-validation was performed on the training set to select the optimal combination of hyperparameters. We selected the hyperparameter corresponding to the highest area under the receiver operating characteristic curve (AUC) for the validation set of each ML model. To develop the SVM model, we used linear, polynomial, sigmoid, and radial kernels as the basis functions; for each kernel, the cost, gamma, degree, and epsilon hyperparameters were used to tune the model. A total of 182,000 combinations of hyperparameters were constructed for the SVM. To develop the RF model, a total of 65,322 combinations of hyperparameters, including ntree, mtry, and nodesize, were used. To develop the XGB model, a total of 163,180 hyperparameters were used, and the optimal hyperparameters comprised eta, gamma, nrounds, and the maximum depth of a tree. To develop the GBM model, a total of 163,180 hyperparameters, including shrinkage, interaction.depth, n.minobsinnode, and bag.fraction, were compared to determine which yielded the highest AUC for the validation set. When developing these four ML models, we performed one-hot encoding for the categorical data and standardized all the continuous features for analysis. Once we developed the final models using the training set, we calculated the AUC and five evaluation metrics, namely, sensitivity, specificity, positive predicted value (PPV), negative predicted value (NPV), F1 score and accuracy, of the test set to measure the predictive ability of each model. To calculate these evaluation metrics, we used the Youden’s index to determine the optimal threshold for classifying the occurrence of AECOPD. Because our focus is on predicting the occurrence of AECOPD as a means to assist patients, higher predictive accuracy and F1 scores were our main priorities. The F1 score (value: 0–1) accounts for both sensitivity and PPV. The formula for the F1 score is

F1 = 2 × (precision × recall)/(precision + recall)(1)

A raincloud plot is a data visualization approach that was employed for summarizing the distribution of AECOPD and the predicted scores; it included individual data points (horizontally jittered), density distributions, and statistical inferences of box plots with medians and interquartile ranges, with whiskers at the 5th and 95th percentiles. The clinical of models was assessed through decision curve analysis. Calibration, which is the agreement between predicted probabilities and observed frequencies of AECOPD, was represented using calibration belts.

The aim of the study was to develop a predictive model to help clinicians with the early detection of risk factors and with decision-making to prevent the occurrence of AECOPD. Therefore, we used SHAP, an explainable artificial intelligence technology, to understand the results of ML model fitting.

All descriptive statistical analyses were performed using SPSS, and the development of the ML models was conducted using R software (version 3.6.2; The Comprehensive R Archive Network: http://cran.r-project.org, accessed on 12 December 2019). Two-sided *p* values < 0.05 were considered statistically significant.

## 3. Results

### 3.1. Study Population Characteristics

A total of 509 patients met the inclusion criteria for analysis, of whom 155 (30.45%) experienced their first AECOPD after enrollment in the COPD P4P program. The patients with AECOPD exhibited poor lung function test results; higher CAT scores; more frequent symptoms of coughing, dyspnea, and wheezing; and a higher prevalence of chronic disease (hypertension, CHF, CPD, sleep disorders, pneumonia, and cancer). The patients with AECOPD were more likely to use COPD medications (such as mono-, dual-, or triple- therapy long-acting bronchodilators and short-acting bronchodilators, antibiotics, oral long-acting bronchodilators, and methylxanthines) and exhibited higher lymphocyte and white blood cell (WBC) counts. The prevalence of AECOPD and distributions of features were similar between the training and test sets, except for the symptom of dyspnea and WBC count. Table 1 displays the selected features used to develop the ML model.

### 3.2. Model Prediction of AECOPD

As indicated in Table 2, the XGB and RF models exhibited moderate discrimination ability (AUC = 0.770 [95% confidence interval (CI) 0.745–0.921] and AUC = 0.751 [95% CI 0.642–0.859], respectively). The GBM and SVM models exhibited higher discrimination ability (AUC = 0.833 [95% CI 0.745–0.921] and AUC = 0.836 [95% CI 0.757–0.915], respectively). The SVM model exhibited the highest sensitivity (82.35%); however, its specificity and PPV for predicting AECOPD were low (69.12 and 57.14%, respectively), and its F1 score (67.47%) and accuracy (73.53%) were insufficient for clinical practice. By contrast, the GBM model exhibited an balanced performance in predicting AECOPD, with a sensitivity of 79.41%, a specificity of 77.94%, and a PPV of 64.29%; its F1 score and prediction accuracy were thus the highest (71.05 and 78.43%, respectively).

**Figure 3:**
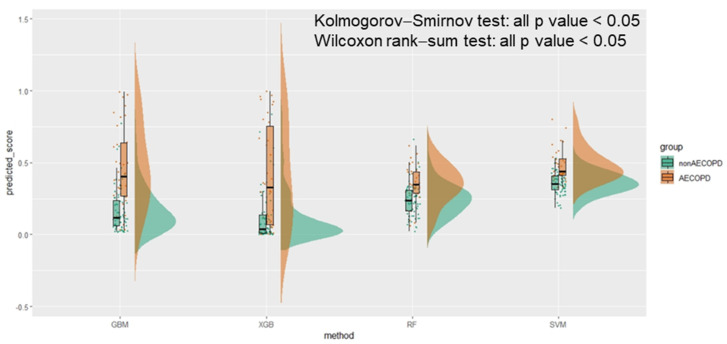
The raincloud plot of AECOPD predicted score in machine learning methods.

The raincloud plot presented in Figure 3 summarizes the distribution of the predicted scores for the AECOPD and non-AECOPD groups. Significant differences were identified between the predicted scores of the groups in all four ML models (all *p* values < 0.001 by the Kolmogorov–Smirnov test), and the median of the AECOPD group was higher than that of the non-AECOPD group (all *p* values < 0.001 by the Wilcoxon rank-sum test). No two boxes in the GBM model overlapped with one another.

**Figure 4:**
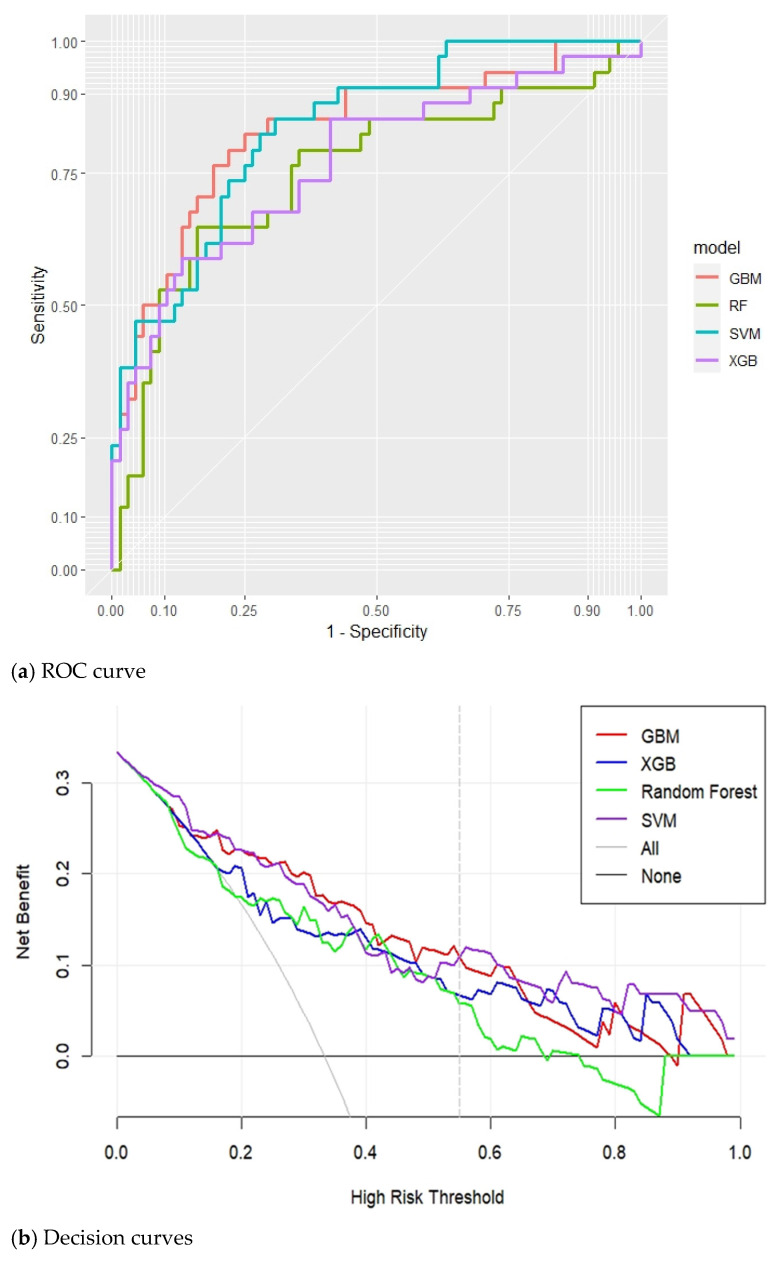
Receiver operation characteristic curves and decision curves of the models for predicting AECOPD. (**a**) Receiver operation characteristic curves (ROC curve); (**b**) Decision curves.

**Figure 5:**
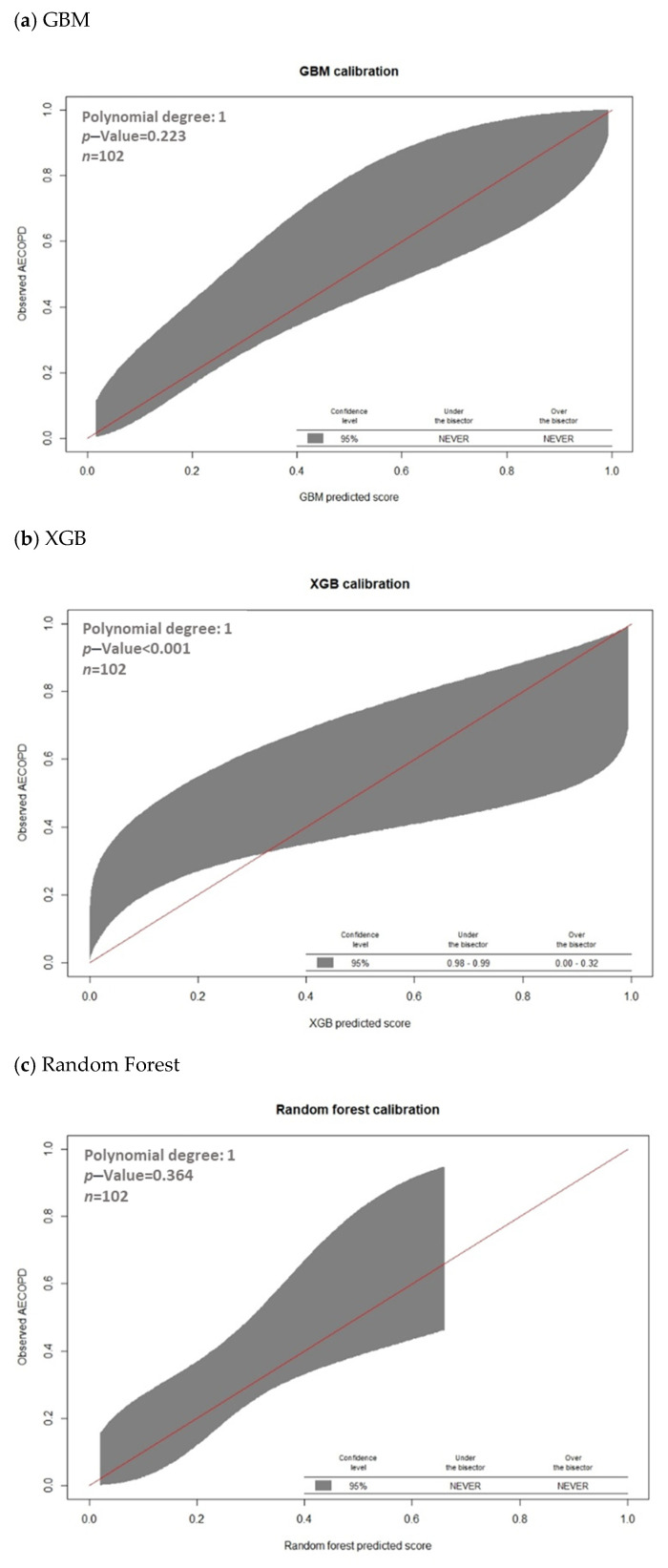

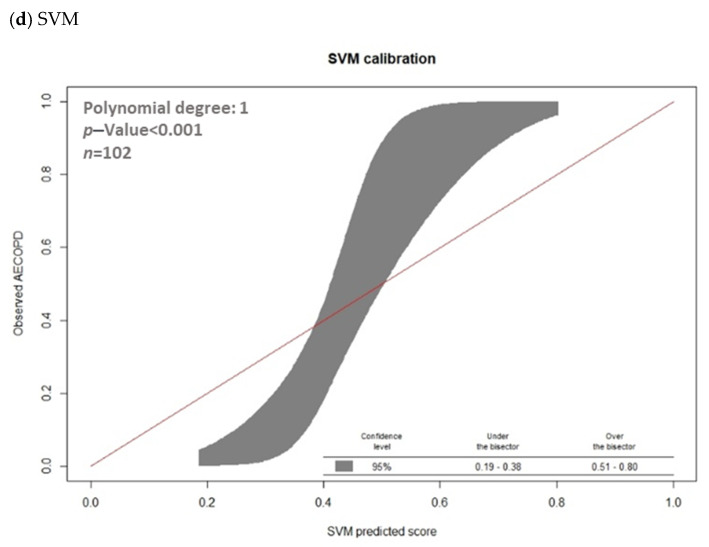
Calibration of machine learning models for predicting AECOPD with calibration belts. (**a**) GBM; (**b**) XGB; (**c**) Random Forest; (**d**) SVM.

The discrimination performance of each of the four ML models, as represented by the receiver operation characteristic and decision curves, is presented in Figure 4. The decision curve analysis revealed that the GBM model exhibited the highest net benefit in distinguishing patients at high risk for AECOPD when the threshold probability was <0.55, whereas the SVM model exhibited the highest net benefit if the threshold probability was >0.55 (Figure 4). Furthermore, the GBM models overlap on the 45° dotted line, indicating good agreement between the ML-predicted and actual probabilities in the calibration plots (*p* value of GBM model = 0.223; Figure 5).

### 3.3. Model Explanations

**Figure 6:**
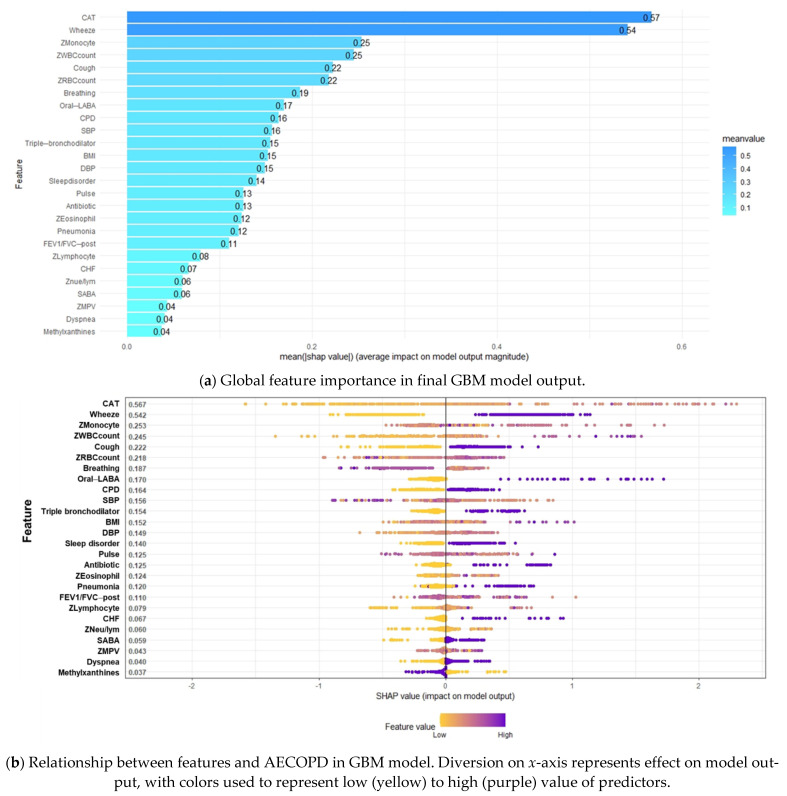
Summary SHapley Additive exPlanations (SHAP) plot. (**a**) Global feature importance in final GBM model output. (**b**) Relationship between features and AECOPD in GBM model.

SHAP values are useful in revealing the contribution of each feature to an individual prediction. Figure 6a presents a plot of features important to the GBM model in order of importance according to the average absolute value of the SHAP values. The beeswarm plot in Figure 6b provides an overview of the effects of individual features on the prediction of AECOPD, with the dots representing the SHAP values of each feature for all individual patients and the colors ranging from yellow (low feature value) to purple (high feature value). The dots are distributed in relation to a vertical line at zero; all the features on the left side of zero exert a negative effect on AECOPD, whereas those on the right side exert a positive effect on AECOPD. The features on the right indicated by purple dots are positively correlated with AECOPD, whereas the features indicated by yellow dots are negatively correlated with AECOPD.

The patients’ CAT scores and symptoms of wheezing were determined to be the two most important features with the highest SHAP values (0.57 and 0.54, respectively; Figure 6a), followed by monocyte count, WBC count, cough, RBC count, breathing rate, oral long-acting bronchodilator use, CPD, SBP, and others. Age, use of dual-therapy bronchodilators, anxiety, cancer, and hypertension did not affect the prediction of AECOPD. As indicated in Figure 6b, higher CAT scores; monocyte, WBC, and RBC counts; BMI; DBP; neutrophil-to-lymphocyte ratio; and eosinophil and lymphocyte counts were positively correlated with AECOPD. The presence of symptoms (wheezing, dyspnea, coughing), chronic diseases (CPD, CHF, sleep disorders, and pneumonia), and COPD medication use (triple-therapy bronchodilators, short-acting bronchodilators, oral long-acting bronchodilators, and antibiotics) were positively associated AECOPD. A high breathing rate, heart rate, and SBP and methylxanthine use were negatively correlated with AECOPD. Although the SHAP value distribution was highly dispersed, the correlations of the features with AECOPD were still consistent with domain knowledge of most of the features.

**Figure 7:**
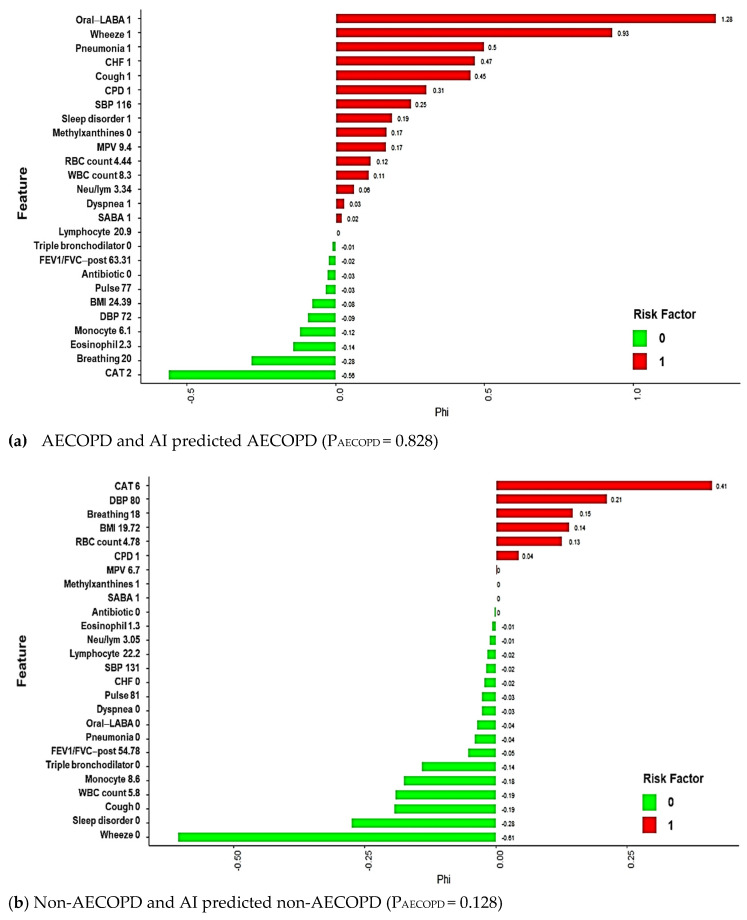

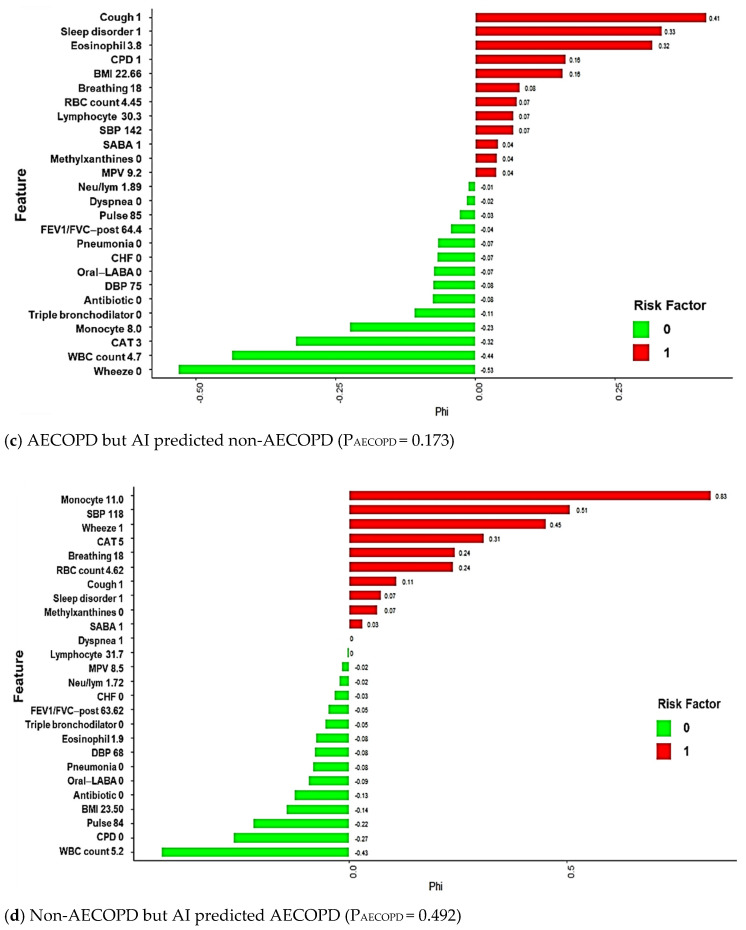
Local explanation plots for individuals with various AECOPD statuses and GBM model predictions. (**a**) AECOPD and AI predicted AECOPD; (**b**) Non-AECOPD and AI predicted non-AECOPD; (**c**) AECOPD but AI predicted non-AECOPD; (**d**) Non-AECOPD but AI predicted AECOPD. Green and red bars correspond to the contribution of the features to the prediction. Green represents a negative value, which decreases the predicted value; Red represents a positive value, which increases the predicted value. *x*-axis represents model prediction value; *y*-axis lists the features and their observed values.

Local explanation results in feature shifts in predictions from base values to model output values. Features that exert and do not exert a significant effect on AECOPD are encoded in red and green, respectively; four local explanation plots of randomly chosen patients are presented in Figure 7. Our proposed model correctly predicted the risk of AECOPD for patients A and B, but incorrectly predicted the risk for patients C and D. Patient A experienced AECOPD, and the ML-predicted probability of AECOPD for patient A was 0.828. The patient’s oral long-acting bronchodilator use; exhibited symptoms (wheezing, dyspnea, coughing); chronic diseases (CPD, CHF, pneumonia, sleep disorders); SBP (116); lab data including Mean platelet volume (MPV) (9.4), RBC count (4.44), and WBC count (8.3); and neutrophil-to-lymphocyte ratio (8.3) were significantly positively associated with AECOPD. By contrast, patient B did not experience AECOPD, and the ML-predicted probability of AECOPD for patient B was 0.128. The patient’s lack of COPD medication use (triple-therapy bronchodilator, short-acting bronchodilator, oral long-acting bronchodilator, and antibiotics), symptoms (wheezing, dyspnea, and cough), and chronic diseases (CHF, pneumonia, sleep disorder), as well as the patient’s regular lab data (MPV; WBC, monocyte, lymphocyte, and eosinophil counts; and neutrophil-to-lymphocyte ratio) contributed to the patient’s relatively low risk of AECOPD.

The predictions for patients C and D are examples of ML mispredictions. The local explanations are presented in Figure 7c,d. The predicted probabilities of AECOPD for the patients were 17.3 and 49.2%, respectively. Although predictions may be incorrect, local explanations can nevertheless provide clinicians with information to increase their awareness of a patient’s condition, which may help prevent AECOPD and address relevant potential risk factors. Furthermore, local explanations are crucial for making personalized health care recommendations.

## 4. Discussion

In this study, we developed and validated an interpretable ML-based risk assessment tool for predicting first-time occurrence of AECOPD. Our findings indicated that the ML models exhibit high discrimination performance, with an average AUC of 0.80. This indicates that ML has the potential for clinical implementation in risk assessment and prediction of first-time AECOPD. Of the ML models, the GBM model achieved the highest performance in this study; therefore, we used this model to develop the interpretable ML-based exacerbation risk assessment tool. Moreover, we used an ML algorithm with a framework based on SHAP and local explanation to assess the key features and establish a modestly accurate model for predicting acute exacerbation in patients with COPD. Providing a visually interpretable feature importance score can help physicians understand the key features of AECOPD in the GBM model, which may support their decision-making processes.

In the outpatient setting, preventing acute exacerbation and avoiding adverse outcomes are the major goals of COPD care. Although a history of AECOPD was determined to be a relatively highly reliable predictor of future exacerbations, it is nevertheless an inadequate basis for a reliable clinical features for informing treatment decisions and methods of AECOPD prevention [18]. Moreover, the discrimination ability of a model that accounted for a history of AECOPD alone was reported to be worse than that of an ML-based model [11]. For example, Tavakoli and colleagues conducted a study that developed an ML-based model to determine which patients were at high risk for hospitalization for AECOPD. Their findings indicated that the GBM model was more effective than a prediction model that employed a history of AECOPD as the only predictor (AUC = 0.82 and 0.68, respectively) [5,17]. A patient history of AECOPD is also not suitable for assessing the risk of first-time AECOPD, and some patient records may lack information regarding prior exacerbations. Hussain et al. developed a GBM prediction model that excluded a history of AECOPD, and the model achieved high discrimination performance, with an AUC of 0.96 [19]. For the framework of the present study, we adopted ML-based modeling as the basis and then incorporated various clinical features using real-world data to account for local population characteristics, and our findings indicated that the GBM exhibited the highest prediction accuracy, with an AUC of 0.83. Similar to the model constructed by Hussain et al., our ML-based model enables the accurate prediction of AECOPD without including exacerbation history as a risk factor. Although our model exhibited lower discriminatory power than did the prediction model developed by Hussain et al., comparing our results to those of their study is difficult because the definition of AECOPD and information about the study population were not included in their paper. Nevertheless, taken together, these results suggest that ML-based models—especially GBM models—are a feasible and accurate method for predicting AECOPD without relying on exacerbation history as the key indicator. These ML-based models have potential to be used as clinical decision-making tools that can help identify patients at high risk of AECOPD who might benefit from adjustment of treatment or referral to a specialist. Moreover, although our GBM model did not include exacerbation history as a feature, the model’s accuracy is comparable with that of previous GBM models that have accounted for exacerbation history. These findings suggest that our prediction model is suitable for assessing a patient’s risk of first-time AECOPD or the risk of patients whose records lack information on prior exacerbations—for instance, patients with new diagnoses of COPD or those with mild COPD.

A recent systematic review of AECOPD prediction models included 27 prediction models that have been developed to account for different variables, such as patient demographics, lung function, symptoms, and COPD risk factors, by using traditional statistical techniques. The models’ performance levels in terms of AUC ranged from 0.58 to 0.78. Compared with traditional statistics, ML provides an alternative method that improves the accuracy of AECOPD prediction models. ML emphasizes performance optimization and is based on a minimum of assumptions about data-generating systems, whereas traditional statistical methods are usually employed to verify specific hypotheses [20]. To compare ML with traditional statistical methods in AECOPD risk assessment, Wang et al. developed AECOPD prediction models by using traditional logistic regression and several ML algorithms, including RF, SVM, logistic regression, k-nearest neighbors, and naïve Bayes algorithms, and the results indicated that the ML-based models achieved higher accuracy [21]. Tavakoli et al. reported that the GBM model exhibited the highest accuracy in predicting AECOPD, exceeding the accuracy of logistic regression, RF, and neural network models [11]. Our findings are consistent with those of previous studies and verified the superior accuracy of the GBM model in AECOPD prediction. These findings indicate that ML-based models, especially GBM models, exhibit high discriminatory accuracy in the prediction of AECOPD.

Feature selection is a key process that involves selecting the optimal subset of features to improve the performance of an ML model. Hussain et al. and Tavakoli et al. developed GBM prediction models that accounted for a range of patient characteristics, namely demographics, vitals, symptoms, questionnaire responses, and laboratory data and demographics, vitals, hospitalizations, and outpatient services and medication dispensation records, respectively. The performance of the prediction models yielded AUCs > 0.80. In our study, the features in the GBM model comprised clinical data, demographics, vital signs, symptoms, prescribed medications, comorbidities, laboratory data, and CAT scores. Compared with previous models, our model included more robust clinical parameters and exhibited comparable performance. In a previous study, asthma exacerbation was predicted on the basis of hundreds of single nucleotide polymorphisms in an RF model [22]. We propose that integrating genomic information into ML models may achieve superior prediction results for AECOPD as well.

COPD is complex and heterogeneous, meaning that it comprises various components with nonlinear dynamic interactions that are not all present in all patients or in a given patient at all times [23]. This dynamic complexity and heterogeneity illustrate the need for a precision medicine approach to improve COPD assessment, treatment, and outcomes [24,25,26]. As ML becomes increasingly integrated into the world of precision medicine, it can help deepen knowledge about the origins and courses of chronic diseases, including COPD [27]. Several studies have reported promising results of using ML approaches in predicting AECOPD. However, black-box logic remains a major obstacle to the application of AI in medicine because a physician will have little to communicate to a patient without an explainable AI model, which may lead to a loss of patient confidence and satisfaction [13]. To overcome this barrier, we applied SHAP and local explanation to improve the predictive effect and interpretability of our model. SHAP is a game-theoretic approach proposed by Lundberg and Lee [28] to interpret feature contribution for a change in the machine learning model (ML) output. The SHAP values reflect the importance of features and provide consistent and locally accurate attribute values for each feature in the prediction model. Using SHAP, we can use it for data visualization, making it easier for users to understand complex black-box integration models. Recently, many studies have been conducted that apply SHAP techniques to various clinical problems, such as coronary artery calcification, venous thrombosis in osteoarthritis patients, and so on [29,30]. To our knowledge, this is the first study to apply SHAP to a GBM black-box integration model. We visually illustrated the interpretability of the complex GBM model by plotting individual risk predictions. Our model indicated that CAT score and wheezing were the most two influential global features with the highest SHAP values, followed by monocyte count, WBC count, coughing, RBC count, breathing rate, oral long-acting bronchodilator use, CPD, SBP, and others. The results of the correlation analysis of the features and AECOPD indicated that higher CAT scores; monocyte, WBC, and RBC counts; BMI; DBP; neutrophil-to-lymphocyte ratios; and eosinophil and lymphocyte counts were positively correlated with AECOPD. Our ranking of the importance of variables broadly corresponded to differences in clinical variables observed in patients with and without AECOPD in our study and is also consistent with previous studies. In addition, local explanation results in feature shifts in the prediction from the base values to the model output values that allows for visual presentation of predicted result to clinical physicians.

Monocyte and oral bronchodilator were selected as key feature for predicting of AECOPD, which are rarely mentioned in their association with AECOPD. For monocytes, despite the mechanism is unclear, the number of monocytes was significantly increased in AECOPD patients compared with healthy subject has been reported in previous study [31]. The evidence supports that monocyte might have the potential role in AECOPD. Although oral bronchodilators are currently rarely. However, it still be selected as a feature for predicting AECOPD by using feature engineering. Moreover, the SHAP value demonstrate that oral bronchodilators are positively correlation with the probability of AECOPD, which means patient who received oral bronchodilator may have high risk of AECOPD. Previous studies indicated that the relative risk of hospitalization was slightly higher in oral bronchodilator compared with inhaled long-acting bronchodilators [32]. There are some factors associated with use of oral bronchodilator in COPD management has been reported. First, although oral bronchodilators are not recommended for COPD treatment, that is except when the use of any inhaled medications is impossible such as patients with disability or patient refuse inhale bronchodilator [33]. Another possibility is that physicians with poor adherence to GOLD guidelines who use oral bronchodilator-based regimens as the mainstay for management of COPD. Taken together, although oral bronchodilator is not recommended as a critical variable in the development of AECOPD, inappropriate use of oral bronchodilators may lead to poor quality of management of COPD, which contribute to AECOPD. Our model provides a history of oral bronchodilators use and its correlation of AECOPD, which can help physicians to review and surveil the inappropriate bronchodilator uses to decrease inadequate oral bronchodilators and optimize inhaled treatments.

The risk prediction features are used to identify the patients with COPD for whom targeted interventions may significantly affect treatment outcomes. In addition, the detailed interpretable information and explainable risk factors included in the results may provide physicians with more insight, helping them to understand personalize the management of patients with COPD; moreover, physicians are not being asked to blindly trust the results of the model. In further application scenarios, our model may not only help physicians determine which patients are at high risk for AECOPD, but also allow physicians to understand the influence of key features that exert a significant effect on a patient’s risk for AECOPD. To our knowledge, our study is the first to establish a personalized explainable ML-based model for predicting AECOPD using local explanations, which are crucial for making personalized health care recommendations for preventing AECOPD.

The predicted probability of AECOPD is around 0.27–0.52 and the local explanations plots with balanced patterns in green and red bars hint at the likelihood of a false positive prediction. When a positive prediction is made, it will prompt physicians to review a patient’s clinical symptom and treatment strategy. Additionally, the registered nurses specialized in COPD care can call the patient a few days after this outpatient clinic to keep concerns about the patient’s condition. Instead, patients will appreciate the caring call and feel like they are being cared for by the clinical facility. Therefore, there is less a threat to patient safety due to the use of inappropriate predictions.

Our study has some limitations. First, we used data from a single health care system, and although the results may be useful for local patients, they may not be generalizable to patients receiving care in other health care institutions. Multicenter external validation is required to optimize the models’ predictive performance. Second, we used only structured data for developing the prediction model in the present study; further research is necessary to investigate the integration of multidimensional data, including unstructured data such as images as well as environmental factors, individual habits, activities, and other factors, to enhance the accuracy of the prediction models. Third, we only used common ML techniques for constructing the prediction model. Recent studies have reported the use of deep learning for constructing medical models, and the establishment of a deep learning model for predicting first-time AECOPD is worth investigating in the future. Fourth, seasonal variation in AECOPD incidence is a common phenomenon, but the rapid changes in temperature were shown to be responsible for AECOPD incidence in two Taiwanese studies [34,35]. COPD patients may be more sensitive to temperature change than the general healthy population, and the trigger for exacerbations is short-term exposures to such temperature changes. However, our current study may not be able to collect real-time data on seasonal temperature changes, which is a limitation of this study.

## 5. Conclusions

In this study, the GBM model was able to accurately evaluate the risk of acute exacerbation in patients with COPD. Applying SHAP and local explanation allowed the model to provide interpretable and visual explanations of individualized risk predictions, which may assist clinical physicians in understanding the effects of key features in the model and in understanding the model’s decision-making process. In conclusion, our model provides an objective and interpretable prediction result that accounts for each patient’s unique characteristics and can help clinicians tailor their treatment of patients with COPD according to the patients’ individualized prognoses.

## Figures and Tables

**Table 1:** Patients’ features in overall cohort and split datasets.

	COPD Patient Data			SPLIT DATA		
	Non-AECOPD (*n* = 354)	AECOPD (*n* = 155)	*p*-Value	Train Data (*n* = 407)	Test Data (*n* = 102)	*p*-Value
Demographic						
Age	72 ± 10	73 ± 10	0.557	73 ± 10	72 ± 11	0.882
BMI	24 ± 4	24 ± 4	0.593	24 ± 4	24 ± 4	0.962
Vital sign						
Pulse	84 ± 13	86 ± 15	0.081	84 ± 14	84 ± 13	0.817
Breathing	19 ± 1	19 ± 1	0.490	19 ± 1	19 ± 1	0.650
SBP	135 ± 18	135 ± 17	0.856	135 ± 18	137 ± 17	0.184
DBP	75 ± 10	76 ± 11	0.110	75 ± 11	77 ± 10	0.072
Lung function						
FEV1/FVC_post	60 ± 10	57 ± 11	0.014	59 ± 11	60 ± 10	0.289
CAT	4 ± 2	6 ± 3	<0.001	4 ± 3	4 ± 3	0.678
Symptoms						
Cough	177(50%)	111(71.6%)	<0.001	230(56.5%)	58(56.9%)	0.949
Dyspnea	142(40.1%)	87(56.1%)	0.001	193(47.4%)	36(35.3%)	0.028
Wheeze	112(31.6%)	93(60%)	<0.001	170(41.8%)	35(34.3%)	0.170
Comorbidity within 1 year						
Chronic pulmonary disease	163(46.2%)	101(65.2%)	<0.001	211(51.8%)	53(52.5%)	0.909
Congestive heart failure	17(4.8%)	17(11%)	0.011	31(7.6%)	3(3%)	0.094
Sleep disorder	112(31.7%)	68(43.9%)	0.008	140(34.4%)	40(39.6%)	0.328
anxiety	26(7.4%)	15(9.7%)	0.378	33(8.1%)	8(7.9%)	0.951
Pneumonia	35(9.9%)	41(26.5%)	<0.001	61(15%)	15(14.9%)	0.973
Hypertension	159(44.9%)	85(54.8%)	0.039	196(48.2%)	48(47.1%)	0.843
Cancer	46(13%)	31(20%)	0.044	63(15.5%)	14(13.9%)	0.685
COPD medication within 6 months						
Short-acting bronchodilators	216(61%)	111(71.6%)	0.022	259(63.6%)	68(66.7%)	0.568
Dual bronchodilator	58(16.4%)	49(31.6%)	<0.001	84(20.6%)	23(22.5%)	0.672
Triple bronchodilator	204(57.6%)	118(76.1%)	<0.001	262(64.4%)	60(58.8%)	0.298
Chronic disease medication within 1 year						
Antibiotic	44(12.4%)	47(30.3%)	<0.001	78(19.2%)	13(12.7%)	0.130
Oral long-acting bronchodilator	14(4%)	25(16.1%)	<0.001	31(7.6%)	8(7.8%)	0.939
Methylxanthines	217(61.3%)	122(78.7%)	<0.001	267(65.6%)	72(70.6%)	0.340
Lab data within 6 months						
WBC count	7.7 ± 3.1	9.1 ± 3.9	<0.001	8.3 ± 3.6	7.4 ± 2.4	0.003
RBC count	4.5 ± 0.7	4.5 ± 0.5	0.165	4.5 ± 0.6	4.5 ± 0.7	0.910
Mean platelet volume	8.1 ± 0.9	8.2 ± 1	0.480	8.1 ± 1	8.1 ± 0.8	0.606
Monocyte	2.6 ± 3.2	2.5 ± 2.7	0.753	9 ± 3.5	8.7 ± 3.8	0.424
Eosinophil	21.9 ± 11.3	20.5 ± 13.2	0.249	2.4 ± 2.5	3 ± 4.4	0.224
Lymphocyte	5.3 ± 7.1	7.2 ± 11.8	0.091	21.7 ± 12.2	20.5 ± 10.7	0.421
Neutrophil/Lymphocyte	8.9 ± 3.5	9 ± 3.7	0.716	5.9 ± 8.6	6 ± 10.1	0.887
Outcome						
AECOPD				121(29.7%)	34(33.3%)	0.479

**Table 2:** Comparison of various models’ performance for predicting AECOPD using test data.

Model	AUC	Threshold	Sensitivity	Specificity	PPV	NPV	F1 Score	Accurate
Gradient boosted machines (GBMs)	0.8326	0.2614	79.41%	77.94%	64.29%	88.33%	71.05%	78.43%
Extreme Gradient Boosting (XGBoost)	0.7703	0.2961	58.82%	86.76%	68.97%	80.82%	63.49%	77.45%
Random Forest (RF)	0.7509	0.3237	64.71%	83.82%	66.67%	82.61%	65.68%	77.45%
Support Vector Machine (SVM)	0.8361	0.3913	82.35%	69.12%	57.14%	88.68%	67.47%	73.53%

The sensitivity, specificity, PPV, and NPV were calculated using Youden’s index.

## Data Availability

The data that support the findings of this study originate from Changhua Christian Hospital clinical research database. Restrictions apply to the availability of these data and they are therefore not publicly available. Due to restrictions, these data can be accessed only by request to Changhua Christian Hospital Big Data center.

## Supplementary Materials

The following supporting information can be downloaded at: www.mdpi.com/article/10.3390/jpm12020228/s1, Figure S1: Feature selection based on random survival forest method and feature importance of selected features in training data. Table S1: Model parameters for the various models.

## Code Availability

The code supporting the results of this research is tailored based on the data, so it is not provided because it cannot be used as a standalone without access to the data itself. The algorithmic models used the standard R code package, which is publicly available.
