# Supplementary material for: Explainable Machine Learning Model for Predicting First-Time Acute Exacerbation in Patients with Chronic Obstructive Pulmonary Disease
Source: J Pers Med. 2022 Feb 7;12(2):228. doi: 10.3390/jpm12020228 (PMC8879653; doi:10.3390/jpm12020228)
Supplement: Supplementary file 1 [file jpm-12-00228-s001.zip › jpm-1577011-supplementary.pdf]

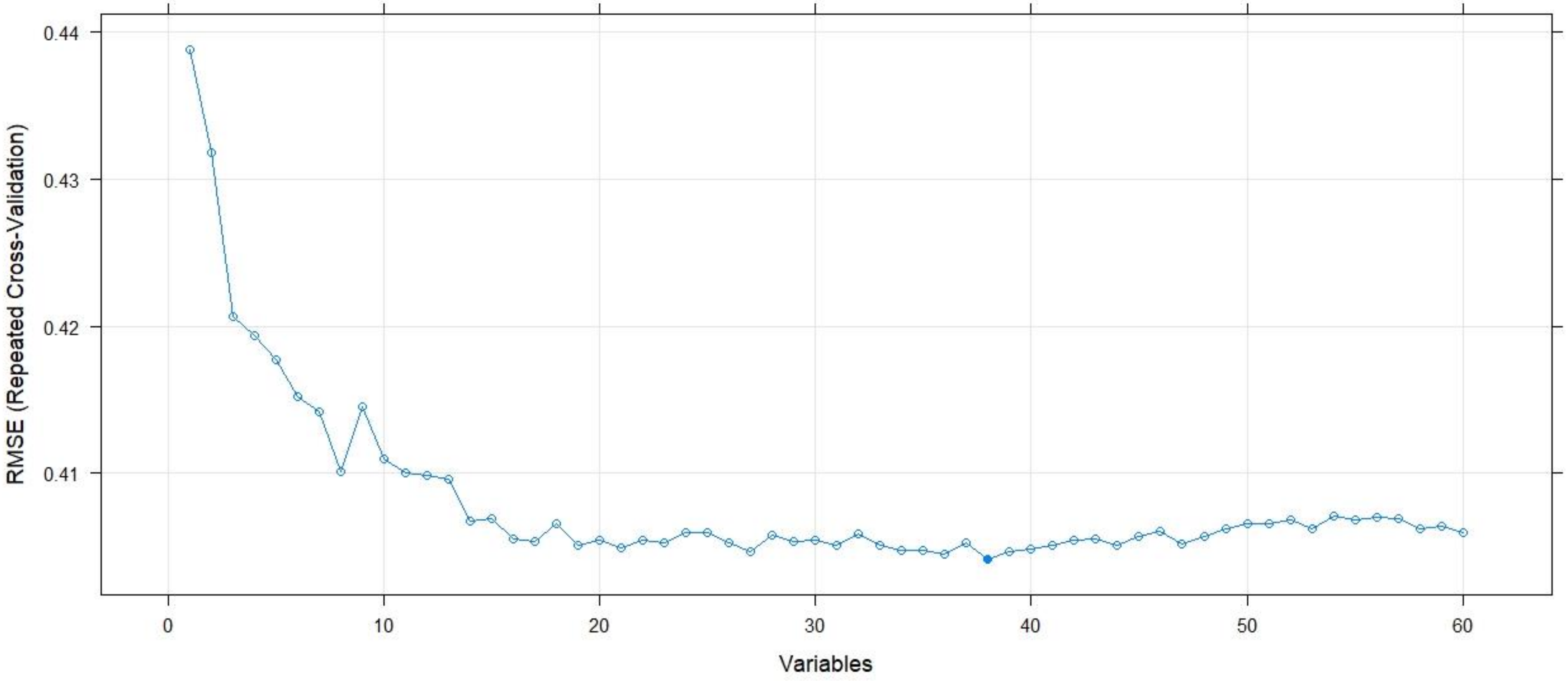

Figure S1. Feature selection based on random survival forest method and feature importance of selected features in training data.

Table S1. Model parameters for the various models

**Model**	**Parameters**
Gradient boosted machines (GBMs)	gbm package:distribution ="bernoulli"<br>n.trees =16<br>shrinkage = 0.39<br>interaction.depth =4<br>n.minobsinnode =5<br>bag.fraction= 0.67
Extreme Gradient Boosting (XGBoost)	xgboost package:<br>objective = "binary:logistic"<br>nrounds =64<br>max_depth =3<br>eta = 0.39
Random Forest (RF)	Random Forest package:<br>num.trees = 71,<br>mtry = 2,<br>min.node.size = 4
Support Vector Machine (SVM)	SVM package:<br>kernel="polynomial"<br>cost=1.01<br>degree=1<br>epsilon=0.9
